# *DPP4* rs17574 polymorphism and elevated DPP4 levels linked to fatty liver in subclinical atherosclerosis: GEA study findings

**DOI:** 10.17305/bb.2025.11950

**Published:** 2025-04-16

**Authors:** Gilberto Vargas-Alarcón, Juan Reyes-Barrera, Guillermo Cardoso-Saldaña, Neftali Antonio-Villa, Giovanny Fuentevilla-Álvarez, José Manuel Fragoso, Rosalinda Posadas-Sánchez

**Affiliations:** 1 Department of Molecular Biology and Research Direction, Instituto Nacional de Cardiología Ignacio Chávez, Mexico City, Mexico; 2 Department of Endocrinology, Instituto Nacional de Cardiología Ignacio Chávez, Mexico City, Mexico; 3 Department of Molecular Biology, Instituto Nacional de Cardiología Ignacio Chávez, Mexico City, Mexico

**Keywords:** Dipeptidyl peptidase-4, DPP4, fatty liver, FL, genotypes, subclinical atherosclerosis, SA, polymorphisms

## Abstract

Dipeptidyl peptidase-4 (DPP4) concentrations are known to correlate with nonalcoholic fatty liver (FL), which is also associated with subclinical atherosclerosis (SA). This study aimed to determine whether DPP4 concentrations and the *DPP4* rs17574 polymorphism are associated with FL in individuals with SA. The study included 378 participants with SA, of whom 143 had FL and 235 did not. DPP4 serum concentrations were measured using a Bioplex system, and *DPP4* rs17574 genotypes were determined using TaqMan assays. Logistic regression was used to assess the relationships between FL, DPP4 concentrations, and rs17574 genotypes. Overall, DPP4 concentrations did not differ significantly between individuals with and without FL. No significant differences in DPP4 levels were observed among *DPP4* genotypes in the total sample. However, within the FL group, significant differences in DPP4 concentration were observed across genotypes: *AA* genotype (134 [106–175] ng/mL), *AG* genotype (128 [114–149] ng/mL), and *GG* genotype (80 [71–117] ng/mL); *P* ═ 0.019. The *DPP4* rs17574 polymorphism was associated with FL under a recessive model (*P* ═ 0.037). DPP4 concentration was also significantly associated with FL: the likelihood of presenting with FL increased by 6.2% for every 10 ng/mL increase in DPP4 levels (*P* ═ 0.009). These findings suggest that DPP4 concentration may serve as a biochemical risk marker for FL in individuals with SA. Moreover, the rs17574 polymorphism may influence DPP4 protein levels, particularly in those with FL. To our knowledge, this is the first study to describe an association between DPP4 concentration, the rs17574 polymorphism, and FL. Assessing DPP4 levels may offer a novel and effective strategy for risk stratification of FL in SA populations.

## Introduction

Nonalcoholic fatty liver disease (NAFLD) is a hepatic condition characterized by the abnormal accumulation of fat in the liver in the absence of significant alcohol consumption. Insulin resistance and certain genetic variants—specifically in the patatin-like phospholipase domain containing 3 (PNPLA3), lipid transporter located on endoplasmic reticulum (TM6SF2), and transmembrane 6 superfamily member 2 genes—have been implicated in the pathogenesis of NAFLD [1–5]. Studies estimate the global prevalence of NAFLD to range from 6% to 35% [6–8]. This condition is associated with a higher prevalence of coronary artery calcification (CAC) across diverse populations [9–11]. A cross-sectional analysis of the Multi-Ethnic Study of Atherosclerosis (MESA) cohort revealed a correlation between NAFLD, subclinical atherosclerosis (SA), and inflammation [12]. Multivariable-adjusted odds ratios (OS) indicated a 1.6-fold increase in the odds of having CAC > 0 among individuals with NAFLD. This association remained significant in White and Hispanic participants. NAFLD is linked to the development of cardiovascular disease (CVD) and atherosclerosis [13]. Recent research has shown a correlation between fatty liver (FL) and CVD, using carotid intima-media thickness (CIMT) as a marker of arterial wall thickening [14, 15], and brachial-ankle pulse wave velocity (BAPWV) as an indicator of arterial stiffness [16–18]. In a study by Keskin et al., patients diagnosed with ST-segment elevation myocardial infarction were classified based on NAFLD severity using ultrasonography. The presence of NAFLD in these patients was associated with adverse clinical outcomes [19]. Patients with FL exhibit endothelial dysfunction [20], a high prevalence of vulnerable coronary plaques [21, 22], and increased coronary artery and abdominal aortic calcification [23]. Zheng et al. [24] identified a significant association between FL and early markers of atherosclerosis, assessed via CIMT and BAPWV. A cross-sectional study demonstrated elevated BAPWV in patients with FL, even after adjusting for age, sex, body mass index (BMI), and lifestyle factors. Furthermore, the study showed an independent association between FL and increased CIMT, even after accounting for conventional cardiovascular and metabolic risk factors [24]. The membrane-bound exopeptidase dipeptidyl peptidase-4 (DPP4), also known as CD26, is expressed in various tissues [25]. Insulin resistance, FL, elevated blood pressure, and oxidative stress have all been linked to DPP4, an enzyme with diverse physiological functions [26–29]. DPP4 plays a role in regulating glucose metabolism, lipid processing, and inflammation, thereby potentially contributing to the progression of atherosclerosis [30, 31]. Elevated DPP4 activity and concentration have been implicated in several metabolic disorders, including obesity, FL, type 2 diabetes mellitus (T2DM), and coronary artery disease (CAD) [32–35]. DPP4 cleaves various substrates, including growth factors, neuropeptides, and chemokines, a function that may enhance its immunomodulatory capabilities. This immune-regulating role is likely mediated through the spleen, the primary organ of systemic immunity [36]. The spleen connects the autonomic nervous system with the circulatory system, and splenic immune cells are known to participate in inflammatory processes associated with both atherosclerotic plaque development and acute myocardial infarction (AMI) [37]. Thus, this section highlights the connection between atherosclerosis and the liver–spleen axis, emphasizing the significant molecular alterations that occur in NAFLD [38]. The DPP4 gene is located on chromosome 2q24.3 and exhibits considerable polymorphism. Variants in this gene are associated with altered DPP4 levels, apolipoprotein B concentrations [39, 40], and increased risk of T2DM [24] and AMI in patients with CAD [41]. Research by our group suggests that the *DPP4* rs17574 G allele may serve as a genetic marker for premature CAD (pCAD) in individuals with diabetes [42]. Given the established associations between DPP4, FL, and CAD, the present study aims to determine whether DPP4 concentration and the *DPP4* rs17574 polymorphism are associated with the presence of FL in individuals with SA.

## Materials and methods

### Subjects

The study included 1500 healthy, asymptomatic participants without a family history of pCAD, selected from the Mexican Genetics of Atherosclerotic Disease (GEA) cohort. All participants were unrelated individuals of Mexican mestizo descent, defined as those born in Mexico with both parents and grandparents also born in the country. Exclusion criteria included congenital heart failure, liver disease, renal disease, thyroid disease, oncological conditions, and pCAD. Participants were recruited from blood bank donors, and formal invitations were disseminated through media outlets and social service centers. To verify participants’ health status, chest and abdominal tomography was performed using a 64-channel multidetector helical computed tomography (CT) system (Somatom Cardiac Sensation 64, Forchheim, Germany). Liver and spleen attenuation were assessed using a single 3-mm tomographic slice at the T11–T12 or T12–L1 vertebral level [43]. Three 1-cm regions of interest were placed on each hepatic lobe and within the spleen parenchyma during image analysis. FL was defined as a liver-to-spleen attenuation ratio (L/S AR) of 1.0 or lower [44]. All imaging procedures were conducted by a single trained observer. To assess consistency, 20 scans were randomly selected and re-evaluated, yielding an intra-observer correlation coefficient of 0.99 (*P* < 0.001). CAC was quantified using the Agatston method [45]. Based on CAC scores, participants were classified into two groups: those with CAC < 0 (individuals with SA) and those with CAC ═ 0 (healthy individuals). Data were also collected on total abdominal fat, subcutaneous abdominal fat, and visceral abdominal fat. The present analysis focused on 378 individuals diagnosed with SA, of whom 143 had FL and 235 did not. Clinical, demographic, biochemical, and anthropometric parameters, along with cardiovascular risk factors, were evaluated as described previously [46–48].

### Determination of DPP4 concentration and polymorphism

DPP4 concentration was measured using a Bio-Plex system, following the manufacturer’s instructions (R&D Systems, Minneapolis, MN, USA). Serum DPP4 levels are expressed in ng/mL. Genomic DNA was extracted from peripheral blood using the QIAamp DNA Blood Mini Kit (QIAGEN, Hilden, Germany). *DPP4* rs17574 genotypes were determined with TaqMan assays on an ABI Prism 7900HT real-time PCR system, according to the manufacturer’s instructions (Applied Biosystems, Foster City, CA, USA).

### Ethical statement

Participants provided written informed consent, and the study complied with the Declaration of Helsinki. The project was approved by the Research Committee of the National Institute of Cardiology Ignacio Chávez (protocol number 18-1082).

### Statistical analysis

We presented data as median (interquartile range), mean ± standard deviation, or frequencies, as appropriate. Continuous variables were compared using either the Mann–Whitney *U* test or the Student’s *t*-test. Categorical variables and Hardy–Weinberg equilibrium were assessed using the chi-square test. Differences in DPP4 serum concentrations were evaluated using the Kruskal–Wallis or Mann–Whitney *U* test. To assess the independent association of *DPP4* rs17574 genotypes with the presence of FL and with DPP4 concentrations, we performed multivariate logistic regression analysis, reporting ORs and 95% confidence intervals (CIs). This association was examined under multiple inheritance models: additive, dominant, heterozygous, recessive, codominant 1, and codominant 2. The statistical power for detecting an association between the *DPP4* polymorphism and FL was 80%, and 87% for the association between DPP4 concentrations and FL. All statistical analyses were performed using SPSS version 15.0 (SPSS Inc., Chicago, IL, USA), with *P* values < 0.05 considered statistically significant.

## Results

### Characteristics of the study population

The current study included 378 control individuals from the overall GEA project population, all exhibiting CAC levels greater than zero. Among these, 143 presented with FL, while 235 did not. The prevalence of FL among patients diagnosed with SA was 37.8%.

Table 1 summarizes the demographic, lifestyle, clinical, and biochemical characteristics, as well as the *DPP4* (rs17574) and *PNPLA3* I148M (rs738409) genotypes, and DPP4 concentrations in individuals with and without FL. Compared to those without FL, patients with FL had significantly higher BMI, increased waist circumference, and elevated levels of triglycerides and CRP (*P* < 0.001), along with greater insulin resistance, as measured by the Homeostasis Model Assessment (*P* < 0.001).

**Table 1:** Characteristics of the studied population

	**Without FL** **(*n* ═ 235)**	**With FL** **(*n* ═ 143)**	P*
Age (years)	59 ± 9	58 ± 7	0.046
Sex (% men)	76	77	0.545
Body mass index (kg/m^2^)	27.8 ± 3.9	30 ± 3.5	<0.001
Waist circumference (cm)	95.2 ± 10.9	100.8 ± 9.3	<0.001
LDL-C (mg/dL)	124 [105–146]	123 [100–142]	0.193
HDL-C (mg/dL)	45 [38–53]	41 [35–45]	<0.001
Triglycerides (mg/dL)	146 [113–194]	176 [137–218]	<0.001
HOMA-IR	3.6 [2.5–5.5]	5.6 [4.2–8.6]	<0.001
DPP4 (ng/mL)	122 [98–152]	128 [105–165]	0.067
High sensitivity CRP (mg/L)	1.3 [0.8–3.0]	2.2 [1.1–3.7]	<0.001
Alanine amino transferase (IU/L)	21 [16–27]	30 [22–42]	<0.001
Aspartate amino transferase (IU/L)	24 [20–28]	27 [23–34]	<0.001
Gamma-glutamyl transpeptidase (IU/L)	26 [19–38]	33 [25–49]	<0.001
Liver to spleen attenuation ratio	1.18 ± 0.12	0.78 ± 0.16	<0.001
Visceral adipose tissue (cm^2^)	157 [116–211]	200 [168–247]	<0.001
CAC Score (Agatston units)	25.1 [5.0–90.8]	21.3 [3.6–81.0]	0.462
Total kilocalories	2154 [1843–2621]	2301 [1896–2814]	0.134
Alcohol consumption (g)	0.7 [0–18]	0.9 [0–2.2]	0.727
Increased VAF (%)	59.1	88.1	<0.001
Type 2 diabetes mellitus (%)	19.1	30.1	0.012
Insulin resistance (%)	53.2	84.6	<0.001
Increased CRP (>3mg/L) (%)	25.0	33.1	0.129
*PNPLA3 I148M genotype (%)*			
*AA*	17.9	16.9	
*AG*	50.6	50.7	0.965
*GG*	31.5	32.4	
*DPP4 rs17574 genotype (%)*			
*AA*	66.8	67.8	
*AG*	30.6	27.3	0.412
*GG*	2.6	4.9	

LDL-C: LDL cholesterol; HOMA-IR: Homeostasis model assessment of insulin resistance; HDL-C: HDL cholesterol; CRP: C reactive protein; CAC: Coronary artery calcification; VAF: Visceral abdominal fat; DPP4: Dipeptidyl peptidase-4; FL: Fatty liver. The values are expressed as mean ± standard deviation, median [interquartile range], o percentage. *Student *t*, Mann–Whitney *U* or chi-square test.

### DPP4 concentrations

Patients with FL had a slightly higher DPP4 concentration (128 [105–165] ng/mL) compared to those without FL (122 [98–152] ng/mL), although the difference was marginal (*P* ═ 0.067).

### Association of the *DPP4* rs17574 polymorphism with FL

The observed and expected frequencies of the *DPP4* rs17574 genotypes were in Hardy–Weinberg equilibrium. As shown in Table 2, under both the recessive and codominant 2 inheritance models, the *GG* genotype of this polymorphism was significantly associated with the presence of FL (recessive model: OR ═ 4.186 [1.092–16.04], *P* ═ 0.037; codominant 2 model: OR ═ 4.346 [1.119–16.99], *P* ═ 0.034). All models were adjusted for age, sex, BMI, triglyceride concentration, T2DM status, total kilocalorie intake, alcohol consumption, and physical activity.

### Association of the *DPP4* rs17574 polymorphism with DPP4 concentration

**Figure 1.**
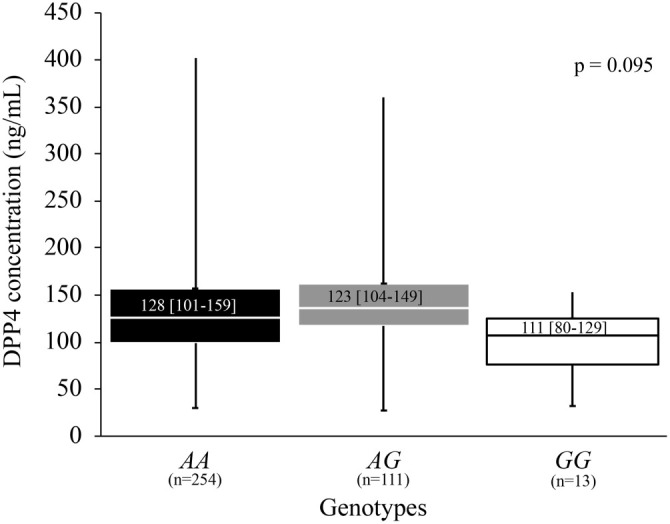
**Association of the *DPP4* rs17574 polymorphism with DPP4 concentration in the whole sample.** People with the *GG* genotype had the lowest levels of DPP4 (111 [80–129]) compared to those with the *AA* (128 [101–159]) and *AG* (123 [104–149]) genotypes. The variations did not reach statistical significance (*P* ═ 0.095). Data shows median and interquartile range. Kruskal–Wallis test. DPP4: Dipeptidyl peptidase-4.

**Figure 2.**
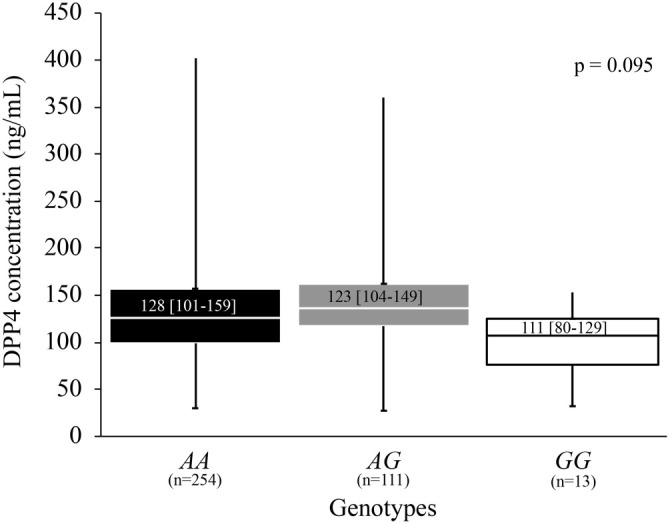
**Association of the *DPP4* rs17574 polymorphism with DPP4 concentration in FL patients.** Patients with the *AG* and *AA* genotypes have higher levels of DPP4 [*AG* genotype (129 [114–149] ng/mL) and *AA* genotype (134 [106–175] ng/mL)] than patients with the *GG* genotype (80 [71–117] ng/mL) (*P* ═ 0.019). Data shows median and interquartile range. Kruskal–Wallis test. DPP4: Dipeptidyl peptidase-4; FL: Fatty liver.

We examined the relationship between DPP4 concentration and rs17574 genotypes by categorizing a sample of 378 individuals into three groups based on genotype. This analysis revealed differences in DPP4 concentrations across the genotypes (*AA*: 128 [101–159], *AG*: 123 [104–149], *GG*: 111 [80–129] ng/mL); however, these differences did not reach statistical significance (*P* ═ 0.095) (Figure 1). When the same analysis was performed separately in individuals with and without FL, statistically significant differences in DPP4 concentration were observed in the FL group (*P* ═ 0.019). In this group, DPP4 levels were as follows: *AA* genotype, 134 (106–175) ng/mL; *AG* genotype, 129 (114–149) ng/mL; and *GG* genotype, 80 (71–117) ng/mL (Figure 2).

**Table 2:** Association of *DPP4* rs17574 polymorphism with fatty liver

**Model**	**OR [95% CI]**	***P***
Additive	1.410 [0.911–2.184]	0.124
Dominant	1.282 [0.771–2.131]	0.339
Recessive	4.186 [1.092–16.04]	0.037
Heterozygous	1.035 [0.613–1.748]	0.898
Codominant 1	1.117 [0.657–1.900]	0.683
Codominant 2	4.346 [1.119–16.99]	0.034

The models were adjusted by age, sex, body mass index, triglycerides concentration, type 2 diabetes mellitus, total kilocalories, alcohol consumption and physical activity. OR: Odds ratio; CI: Confidence interval.

**Figure 3.**
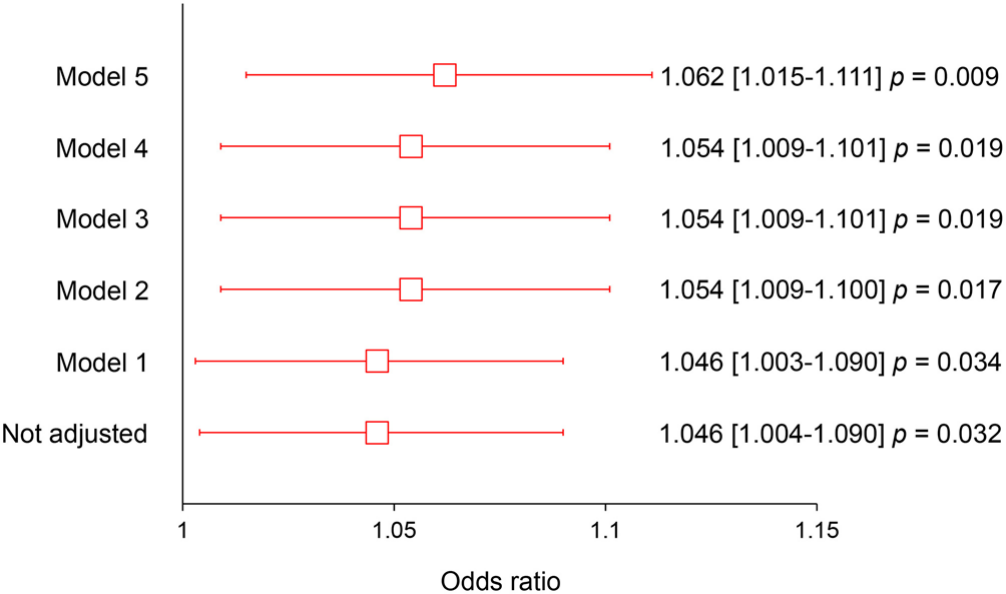
**The association between DPP4 concentration and FL.** The models were adjusted based on different factors related to FL, such as the *PNPLA3* I148M genetic marker that is linked to the risk of developing FL. Model 1: Adjusted by age and sex; Model 2: Adjusted by model 1, BMI and triglycerides concentration; Model 3: Adjusted by model 2, kilocalorie consumption and grams of alcohol; Model 4: Adjusted by model 3, physical activity and T2DM; Model 5: Adjusted by model 4, *DPP4* (rs17574) and *PNPLA3* I148M (rs738409) genotypes. When the more adjusted model (*P* ═ 0.009) is looked at, a rise of ten ng/dL in DPP4 levels is linked to a 6.2% increase in the chance of having FL. DPP4: Dipeptidyl peptidase-4; FL: Fatty liver.

The association between DPP4 concentration and FL was further explored using logistic regression, adjusting for various covariates. The analysis also included an adjustment for the PNPLA3 polymorphism, which is associated with an increased risk of developing FL in several populations, including the Mexican population [47, 49–52]. Model 1: Adjusted for age and sex (OR ═ 1.046 [1.003–1.090], *P* ═ 0.034). Model 2: Adjusted for age, sex, BMI, and triglyceride concentration (OR ═ 1.054 [1.009–1.100], *P* ═ 0.017). Model 3: Included age, sex, BMI, triglyceride concentration, total kilocalories, and alcohol consumption (OR ═ 1.054 [1.009–1.101], *P* ═ 0.019). Model 4: Added physical activity and T2DM to model 3 (OR ═ 1.054 [1.009–1.101], *P* ═ 0.019). Model 5: Fully adjusted for age, sex, BMI, triglyceride concentration, total kilocalories, alcohol consumption, physical activity, T2DM, and the *DPP4* (rs17574) and *PNPLA3* I148M (rs738409) polymorphisms (OR ═ 1.062 [1.015–1.111], *P* ═ 0.009). In the fully adjusted model (Model 5), a 10 ng/dL increase in DPP4 concentration was associated with a 6.2% increase in the likelihood of presenting with FL (*P* ═ 0.009) (Figure 3).

## Discussion

This study is the first to evaluate the association and interaction between DPP4 concentration and the *DPP4* rs17574 polymorphism with the presence of FL in individuals with SA. Although no overall differences in DPP4 concentration were observed between patients with and without FL, stratification by *DPP4* genotypes revealed that patients with FL exhibited different DPP4 concentrations depending on their genotype. Specifically, individuals with the *AA* genotype had higher concentrations of the molecule. The *DPP4* rs17574 polymorphism was associated with the presence of FL, with the likelihood of developing FL increasing by 6.2% for every 10 ng/dL rise in DPP4 concentrations. These associations were independent of cardiometabolic risk factors and the *PNPLA3* I148M (rs738409) polymorphism. The *PNPLA3* I148M variant is the genetic marker most commonly linked to the development of FL worldwide, including among Hispanic populations [42, 49–52]. While FL has been associated with various genetic polymorphisms, several studies have highlighted the roles of *PNPLA3*, *SAMM50*, *NCAN*, *GCKR*, and *LEP* in susceptibility to NAFLD [53–56]. In our study, we adjusted for variation in the *PNPLA3* gene—the most prevalent genetic factor related to FL globally [47, 49–52]. The other genes were excluded from our analysis due to their associations being limited to specific populations.

FL is a chronic pathological condition characterized by excessive triglyceride accumulation in hepatocytes and has become one of the most prevalent liver diseases worldwide [57]. Its emergence is strongly associated with metabolic disorders, and its incidence has risen markedly in recent decades, paralleling the global increase in obesity and T2DM [58]. Additionally, FL is independently associated with SA, a condition marked by increased CIMT [24].

DPP4 plays a multifaceted role, influencing not only diabetes and glucose metabolism but also affecting cardiovascular health and potentially contributing to heart diseases such as atherosclerosis [59–61]. The gene encoding this enzyme is highly polymorphic, with certain variants linked to the development of several diseases, including CVD [62]. Previous genetic studies have shown associations between *DPP4* polymorphisms and serum lipid levels [63]. Specifically, the rs3788979 variant in the *DPP4* gene has been associated with an increased risk of AMI in individuals with established CAD [63]. Our research group previously reported that, in the Mexican population, patients with pCAD and T2DM exhibit the lowest serum concentrations of DPP4. Among individuals with T2DM, carriers of the rs17574 *G* allele demonstrated a 30% reduced risk of developing pCAD, accompanied by lower DPP4 levels. In diabetic patients, this polymorphism may act as a genetic marker offering protection against pCAD [42]. Additionally, this allele has been associated with a reduced risk of hypoalphalipoproteinemia, insulin resistance, and hyperinsulinemia, along with decreased serum DPP4 levels [64].

**Figure 4.**
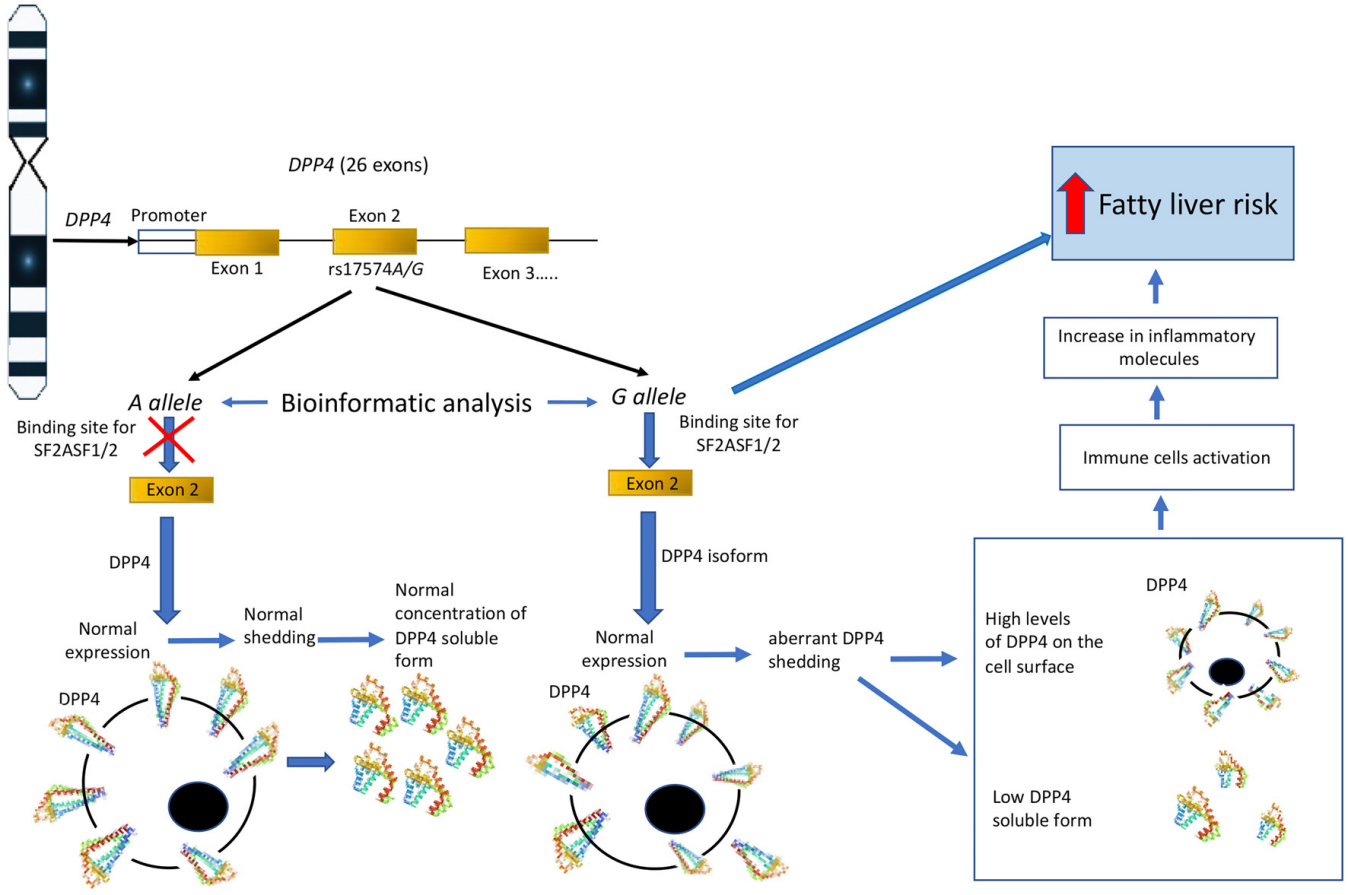
**Hypothesis of DPP4 participation in the development of fatty liver.** When the *G* allele is present, it creates sites for the SF2ASF1 and SF2ASF2 splicing regulatory proteins to bind to exonic splicing enhancers. The introduction of this ESE site alters the local splicing landscape by enhancing the interaction efficiency of the regulatory proteins, which shifts the balance toward the production of alternative DPP4 mRNA isoforms. These isoforms would have a normal expression on the cell surface but an aberrant shedding. As a result, the production of soluble forms would decrease, while the majority of the molecule would stay anchored to the membrane. The membrane-anchored form can act as a costimulatory molecule for T-cell activation and promote inflammation that would increase the risk of FL. DPP4: Dipeptidyl peptidase-4; FL: Fatty liver.

The *DPP4* rs17574 polymorphism associated with FL in individuals with SA is located in exon 2 of the gene. Bioinformatic analyses revealed that the *G* allele at this position creates a novel binding site for the splicing regulatory proteins SF2/ASF1 and SF2/ASF2. These serine/arginine-rich splicing factors bind to exonic splicing enhancers (ESEs) within exon 2, promoting the recruitment of spliceosomal components necessary for exon recognition and inclusion. The introduction of this ESE site alters the local splicing landscape by enhancing SF2/ASF protein binding, thereby shifting the balance toward the production of alternative *DPP4* mRNA isoforms. These isoforms may differ in their inclusion or exclusion of exonic regions critical for post-translational modifications and proteolytic processing. Structurally, the resulting DPP4 protein exhibits alterations in its extracellular domain—particularly in the flexible stalk region, which is responsible for protease cleavage and shedding. Multiple *DPP4* isoforms have previously been detected in human plasma and placenta [65, 66], as well as in both normal and cancerous lung tissue [67, 68]. DPP4 is ubiquitously expressed on the surface of many cell types and also exists in a soluble form in the circulation [69], performing dual roles as both membrane-bound and soluble isoforms [70, 71]. Soluble DPP4 is generated via a non-classical secretory mechanism involving proteolytic cleavage in the flexible stalk region, producing a circulating form that retains enzymatic activity similar to its membrane-bound counterpart [71, 72–75]. Membrane-bound DPP4 interacts with key molecules such as adenosine deaminase and contributes to T cell activation, thereby amplifying pro-inflammatory signaling pathways [76]. Recent studies have reported an increased presence of CD8+ lymphocytes in visceral adipose tissue, where DPP4 (CD26) is highly expressed on the cell surface [77]. This expression facilitates macrophage recruitment and drives inflammation within the tissue [78]. In hepatic tissue, this inflammatory environment promotes insulin resistance and disrupts lipid metabolism. Collectively, these molecular changes accelerate the progression of hepatic steatosis, establishing a mechanistic link between the rs17574 *G* allele, abnormal splicing, and a heightened risk of FL and related metabolic diseases (Figure 4).

A key strength of this study is the inclusion of a cohort of Mexican individuals with SA, encompassing both those with and without FL. We performed a comprehensive evaluation using imaging techniques, clinical assessments, and laboratory analyses, all conducted in accordance with standardized protocols. This approach enabled effective control of confounding factors that might influence the outcomes. To our knowledge, this is the first cohort in Mexico to be studied for the coexistence of these conditions. However, several limitations should be considered. First, as the GEA study is observational, the current findings do not establish a causal relationship between DPP4 concentrations, the rs17574 genetic variant, and the presence of FL. A distinctive aspect of the GEA study design is that the control group was selected exclusively from individuals without a personal or family history of pCAD, which may limit the generalizability of the results to the broader population. Regarding FL diagnosis, multiple noninvasive techniques are available. Magnetic resonance spectroscopy (MRS) is the most sensitive method, capable of detecting liver fat accumulation as low as 5%. However, MRS remains costly and is not widely accessible. Ultrasonography offers a more affordable and straightforward alternative but is operator-dependent and provides only qualitative information. CT, while less sensitive to mild steatosis—detecting FL in cases where fat accumulation exceeds 30%—offers greater reproducibility and specificity compared to ultrasound [43]. Due to ethical considerations, liver biopsies were not performed to confirm FL diagnosis. Nonetheless, previous studies have demonstrated a significant correlation between CT-derived liver attenuation values and the histological grading of steatosis [79].

## Conclusion

This study demonstrates that each 10 ng/mL increase in DPP4 concentration is associated with a 6.2% increased probability of developing FL in subjects with SA. The *DPP4* rs17574 *GG* genotype is linked to the onset of FL in these patients. Among individuals with FL, those carrying the *AG* or *AA* genotypes exhibit higher DPP4 concentrations compared to those with the *GG* genotype. This is the first study to report on the interaction between DPP4 concentration and the *DPP4* rs17574 polymorphism, as well as its association with FL. The findings suggest that DPP4 concentration may serve as a biochemical risk marker for the presence of FL in individuals with SA, and its measurement could represent a novel and effective approach for risk stratification. However, further research evaluating the sensitivity, specificity, and predictive value of this marker is needed to validate these results. Understanding the *DPP4* rs17574 variation and its influence on DPP4 levels may support the development of improved, more individualized treatments—an important step toward advancing personalized medicine.

## Data Availability

Data of this study are accessible upon requirement to the author of correspondence.
